# Enhancing prediction of primary site recurrence in head and neck cancer using radiomics and uncertainty estimation

**DOI:** 10.3389/frai.2025.1623393

**Published:** 2025-09-08

**Authors:** Yu Hu, Kimberly Taing, Jing Wang, David Sher, Michael Dohopolski

**Affiliations:** Medical Artificial Intelligence and Automation Lab, Department of Radiation Oncology, UT Southwestern Medical Center, Dallas, TX, United States

**Keywords:** head and neck cancer, medical image analysis, machine learning, feature and model selection, SAM-Med3D, test-time augmentation, conformal prediction

## Abstract

**Introduction:**

Head and neck squamous cell carcinomas (HNSCC) present a significant clinical challenge due to high recurrence rates despite advances in radiation and chemotherapy. Early detection of recurrence is critical for optimizing treatment outcomes and improving patient survival.

**Methods:**

We developed two artificial intelligence (AI) pipelines—(1) machine learning models trained on radiomic and clinical data and (2) a Vision Transformer-based model directly applied to imaging data—to predict HNSCC recurrence using pre- and post-treatment PET/CT scans from a cohort of 249 patients. We incorporated Test-Time Augmentation (TTA) and Conformal Prediction to quantify prediction uncertainty and enhance model reliability.

**Results:**

The machine learning models achieved an average AUC of 0.820. The vision transformer model showed moderate performance (AUC = 0.658). Uncertainty quantification enabled the exclusion of ambiguous predictions, improving accuracy among more confident cases.

**Discussion:**

Our machine learning models achieved strong performance in predicting HNSCC recurrence from radiomic and clinical features. Incorporating uncertainty quantification further improved predictive performance and reliability.

## 1 Introduction

Head and neck squamous cell carcinomas (HNSCC) account for nearly 4% of all new cancer diagnoses in the United States, with over 60,000 new cases reported annually and a 25% mortality rate (Pfister et al., 2020; Shen et al., 2015). Radiation therapy is a cornerstone of HNSCC treatment, often combined with chemotherapy depending on tumor type and stage (Pfister et al., 2020; De Felice et al., 2023). Although recent therapeutic advances have improved survival (Guo et al., 2021), recurrence remains a serious challenge: ~10% of early-stage HNSCCs recur within the first year of treatment (Borsetto et al., 2021), and 30%–40% of advanced-stage cancers eventually relapse (5). Risk factors for recurrence include tumor subtype, treatment modality, disease stage, age, and comorbidities (Leeman et al., 2017; Haring et al., 2023). Given these complexities, accurately predicting and detecting tumor recurrence is crucial for guiding more personalized clinical decisions after radiation therapy. Early detection allows low-risk patients to avoid unnecessary follow-up scans, while high-risk patients can benefit from more aggressive treatments or closer monitoring.

Standard methods for detecting recurrence include clinical examination and endoscopy for mucosal lesions, while 18F-fluorodeoxyglucose positron emission tomography/computed tomography (FDG-PET/CT) is preferred for deeper tissue evaluation (Gule-Monroe et al., 2020; Pfister et al., 2020). Conducted at least 12 weeks post-treatment, FDG-PET/CT remains the first-line surveillance tool for detecting residual or recurrent disease (Gule-Monroe et al., 2020; Cheung et al., 2016). Early detection of recurrence using FDG-PET/CT or other types of advanced imaging is critical for improving patient outcomes, as it allows for prompt intervention by clinicians. This can potentially reduce the need for aggressive treatments, as well as improve survival rates while minimizing unnecessary treatments for low-risk patients. As a result, it would not only reduce side effects but also optimize use of healthcare resources. Furthermore, early detection and intervention can prevent disease progression, lowering the disease burden on patients and thus leading to a better overall quality of life. However, false-positive and false-negative findings from the imaging can undermine early detection. To address these limitations, radiomics has emerged as a promising adjunctive approach for improving surveillance accuracy and treatment plans. However, false-positive and false-negative findings can undermine early detection.

Radiomics involves extracting quantitative features from medical imaging, such as CT and PET scans, many of which are not readily visible to the human eye. These features can reveal critical information about tumor heterogeneity and patient response to treatment. By applying advanced computational techniques and machine learning, this imaging data is transformed into models that can predict disease outcomes, such as recurrence. In doing so, radiomics can optimize personalized treatment planning by offering a more detailed and objective understanding of the disease (Van Timmeren et al., 2020).

Numerous studies have demonstrated radiomics' potential for predicting recurrence and treatment outcomes in HNSCC with encouraging results (Tortora et al., 2023; Zhang et al., 2022; Wang K. et al., 2023). For instance, Gangil et al. (2022) found that integrating clinical and radiomics data using Kernel Support Vector Machine (KSVM) significantly enhanced predictive accuracy compared to clinical or radiomics data alone, improving the ability to predict distant metastases, locoregional recurrences, new primaries, and residual disease. Similarly, Wang K. et al. (2023) showed that models built with delta-radiomics features—combining clinical and radiomics data—improved locoregional recurrence prediction accuracy. Fh et al. (2021) developed models for HNSCC recurrence using radiomics data from both the planning target volume (PTV) and gross tumor volume (GTV), achieving high sensitivity, specificity, and accuracy, demonstrating that incorporating features from multiple regions of interest can further enhance prediction accuracy. Additionally, Oka et al. (2024) demonstrated the power of combining radiomics with Gaussian noise upsampling (GNUS), which improved both sensitivity and specificity for predicting recurrence. These studies illustrate radiomics' capacity to extract meaningful insights from imaging data.

However, a critical yet underexplored dimension of predictive modeling in oncology is uncertainty quantification—the ability to assess how confident the model is in each individual prediction. Techniques such as TTA and conformal prediction offer principled methods to measure and control this uncertainty. While prior studies have applied methods like data augmentation or ensemble learning for robustness, the explicit integration of formal uncertainty frameworks in radiomics-based recurrence prediction remains rare. Furthermore, few studies leverage both TTA and conformal prediction in tandem or systematically assess how filtering uncertain predictions may impact overall diagnostic accuracy.

To address this gap, our study integrates both TTA and conformal prediction into radiomics-based recurrence models using a relatively large and paired PET/CT dataset of over 200 patients. By identifying and excluding high-uncertainty cases, we aim to improve model robustness and interpretability, particularly in detecting local recurrence. In this paper, we evaluate the impact of uncertainty-guided filtering on model performance, and demonstrate how uncertainty estimation can enhance the clinical reliability of radiomics models for HNSCC recurrence prediction.

## 2 Methods

We sought to develop artificial intelligence (AI) models to predict the local recurrence of head and neck cancer using pre and post treatment PET/CT imaging. We developed two AI pipelines: (1) machine learning models trained on tabular data combining extracted radiomic features with clinical features, and (2) a vision transformer-based model applied directly to the imaging data. The performance of the two pipelines was compared. Finally, uncertainty analysis was performed to evaluate prediction robustness.

### 2.1 Patient demographics

This study included 322 patients with pre-treatment CT and PET images and 331 patients with post-treatment CT and PET images. Of these, 249 patients had paired pre- and post-treatment images. Treatments were administered between 2005 and 2020, with 96.7% of patients treated between 2007 and 2015. The cohort was predominantly male (79.5%). The most common cancer histology was squamous cell carcinoma (95.6%), and the primary disease site was the oropharynx (68.3%). Detailed demographic characteristics are provided in Supplementary Tables S1, S2.

### 2.2 Classical machine learning models

The machine learning pipeline consisted of feature selection to reduce dimensionality, model development with threshold optimization for class imbalance, model selection to determine the optimal fold-wise classifier, and evaluation to assess final predictive performance. Prediction uncertainty was assessed using TTA (Wang K. et al., 2023) and Conformal Prediction (Molnar, 2023). Model performance was evaluated using metrics such as Area Under the Receiver Operating Characteristic Curve (AUC), sensitivity, and specificity.

#### 2.2.1 Data preparation and partitioning

A total of 107 radiomic features were extracted from the pre- and post-treatment CT and PET images using the Pyradiomics library (Van Griethuysen et al., 2017). Additionally, 15 clinical features, including demographic, staging, and treatment-related information, were obtained from medical records, resulting in a combined dataset of 107 × 4+15 = 443 features.

The distribution of the outcome variable (local recurrence) was imbalanced, with 22.9% positive cases and 77.1% negative cases. We addressed this imbalance using threshold optimization, described in detail below.

We employed five-fold cross-validation. This technique partitions the available dataset into five equally sized folds. During each of the five iterations of the cross-validation process, one fold is held out as the validation set, one fold is held out as the test set, while the remaining three folds are used for training the model. The process is repeated five times, such that each fold serves as the validation and test set exactly once.

#### 2.2.2 Feature selection

To address high dimensionality and improve model generalizability, we applied a two-stage feature selection procedure focused on radiomic features, while retaining all clinical variables.

First, correlation-based filtering was applied to reduce redundancy among radiomic features (Teng et al., 2022). Pearson correlation coefficients were computed between all feature pairs, and any pair with a correlation coefficient >0.7 was considered highly correlated. For each such pair, the feature with the higher average absolute correlation to all other features was removed. This step typically reduced the 428 radiomic features to <100.

Next, the Least Absolute Shrinkage and Selection Operator (LASSO) was used to identify the most predictive subset of radiomic features. By adjusting the regularization strength, we generated multiple candidate feature sets containing between six and 10 radiomic variables. These sets were then evaluated by training a Support Vector Classifier (SVC) on the training data and computing the Youden Index on the validation set. The feature set achieving the highest validation Youden Index was selected for downstream model development.

Finally, each selected radiomic feature set was combined with the full set of 15 clinical variables, yielding a final set of ~21–25 features per fold. All feature selection steps were performed using only training and validation data within each fold.

#### 2.2.3 Model development and evaluation

We constructed five candidate classifiers for each fold: Logistic Regression, SVC with linear and polynomial kernels, Explainable Boosting Classifier (EBC), and eXtreme Gradient Boosting (XGBoost). All models were trained on the selected feature set, which included all clinical variables and the radiomic features selected via LASSO.

For each model, threshold optimization was performed to determine the probability cutoff that maximized the Youden Index on the validation set. Rather than defaulting to 0.5, we evaluated thresholds between 0 and 0.5 in small increments 0.01, selecting the value that yielded the best trade-off between sensitivity and specificity. This validation-derived threshold was then applied to the test set in the corresponding fold.

To identify the best-performing model within each fold, for each model, we performed training on the designated training data and evaluated its performance on the validation set. The Youden Index (Sensitivity + Specificity – 1) was used as the primary selection criterion to identify the most balanced and clinically relevant classifier. Among all candidate models, the one achieving the highest Youden Index on the validation set was selected as the optimal model for that fold.

**Figure 1:**
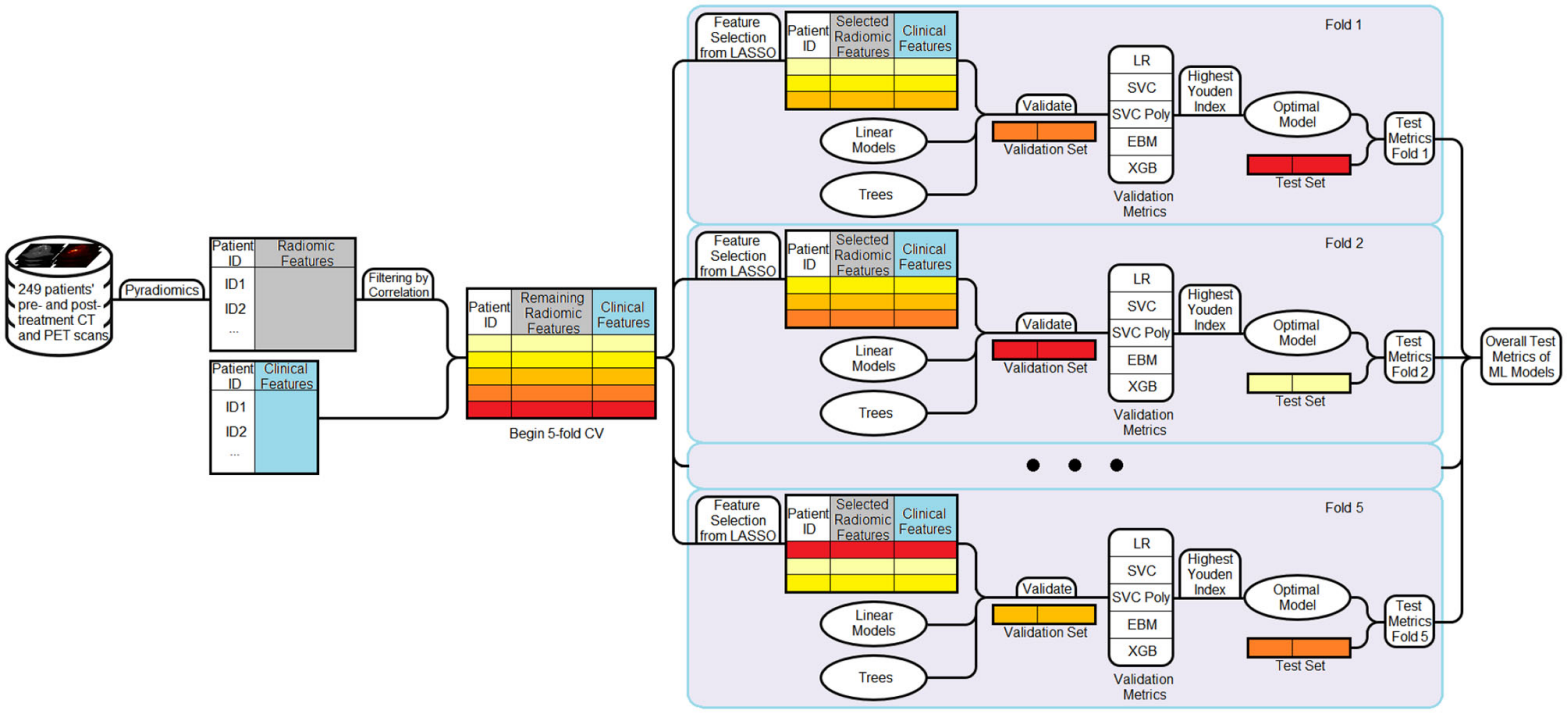
Workflow of the machine learning pipeline. Radiomic and clinical features are combined to form the dataset. Radiomic features are first filtered using a correlation-based method (threshold = 0.7). A five-fold cross-validation strategy is then employed. In each fold, LASSO is applied to the filtered radiomic features from the training set to select 6–10 informative variables, which are then combined with the full set of clinical features. Multiple models are trained and evaluated on the validation set, and the model achieving the highest Youden Index is selected as the optimal model for that fold. This model is then evaluated on the corresponding test set. Performance metrics are aggregated across all folds to obtain final results.

The chosen model was then evaluated on the test set, and its predictions were recorded. This process was repeated across all five folds. To summarize overall performance, predictions from the test sets of all folds were aggregated. Performance metrics, including AUC, accuracy, sensitivity, specificity, positive predictive value (PPV), negative predictive value (NPV), and PRAUC, were reported for the final evaluation. The full machine learning workflow is illustrated in Figure 1.

#### 2.2.4 Evaluating variability across random states

To assess how random states affect data splitting and model initialization, we used a predefined list of random states. The model was run for each random state, and the test metrics were collected to evaluate variability in the results. Averaging these metrics provides a more reliable assessment of the model's overall performance compared to relying on a single data split, while the standard deviation of the metrics offers insight into the model's stability.

### 2.3 Vision transformer-based model

Transformer architectures have recently shown promising performance in computer vision tasks through self-attention mechanisms that capture both local and global contextual information (Dosovitskiy et al., 2020). Vision Transformers are transformer models that process image data as sequences of patches, using self-attention mechanisms to capture spatial relationships. In this study, we adopted SAM-Med3D (Wang H. et al., 2023), a ViT-based architecture adapted from the Segment Anything Model (SAM) for volumetric medical image analysis. SAM-Med3D comprises three primary components: an image encoder that processes 3D image volumes, a prompt encoder for optional user-defined inputs (e.g., points or bounding boxes), and a mask decoder that generates segmentation outputs. We used only the image encoder as a feature extractor to enable downstream binary classification.

#### 2.3.1 Model implementation details

For each patient, we used paired pre- and post-treatment CT and PET images. These four volumes were arranged along the channel dimension, forming a 4-channel input with a shape of [*B*, 4, 64, 64, 64], where *B* is the batch size. All images were cropped to the smallest bounding box encompassing the primary tumor site and then resized to 64 × 64 × 64 via center cropping or zero-padding. Voxel intensities were min-max normalized using the 0.5th and 99.5th percentiles computed from the training dataset to reduce the influence of outliers.

We used the pretrained SAM-Med3D model (Wang H. et al., 2023), which is based on the Vision Transformer-Base (ViT-B) backbone adapted for 3D medical imaging tasks. In our pipeline, we used the image encoder as a fixed feature extractor and appended a lightweight binary classification head consisting of a global average pooling layer followed by a fully connected layer with sigmoid activation. To enable effective transfer learning, we partially fine-tuned the SAM-Med3D encoder by freezing.

The model was trained for 70 epochs using the AdamW optimizer with an initial learning rate of 2 × 10^−4^, weight decay of 0.002, and a OneCycleLR scheduler with a peak learning rate of 2 × 10^−3^. We used binary cross-entropy with logits as the loss function. A batch size of 8 was used for all experiments. To address class imbalance in the dataset, we applied weighted random sampling with a target ratio of 60:40 between negative and positive cases.

To improve robustness during training, random augmentations were applied to each input volume, including affine transformations (rotation and translation) with a probability of 0.4 and random flips along the coronal and sagittal planes with a probability of 0.3. All preprocessing and augmentation steps were implemented using the TorchIO library. The model was trained and evaluated using the PyTorch Lightning framework, and results were averaged across five-fold cross-validation.

### 2.4 Uncertainty analysis

We assessed and compared prediction uncertainty across different cohorts using TTA and Conformal Prediction.

#### 2.4.1 TTA

TTA was implemented by introducing variability to the input data during inference. Specifically, Gaussian noise was added to the radiomic features five times per sample, with a magnitude of 0.1 × the variable-wise standard deviation—deliberately higher than the noise used during training. The model generated predictions for each augmented version, and the final output for each sample was computed by averaging the predicted probabilities. Labels remained unchanged throughout the process.

To quantify prediction uncertainty, we computed an off-centered entropy score from the averaged predicted probabilities for each sample (Guermazi et al., 2018; Lenca et al., 2010). Let *p* denote the predicted probability of the positive class, and let τ∈(0, 1) be the optimal decision threshold determined on the validation set in a given fold, the off-centered entropy shifts the maximum entropy from *p* = 0.5 to *p* = τ, aligning the uncertainty peak with the decision boundary actually used during evaluation. This adaptation provides a more accurate measure of predictive uncertainty, particularly in settings where class imbalance or threshold optimization justifies using τ≠0.5. The mathematical formulation is detailed in the Supplementary material.

We then selected entropy values corresponding to the 70th, 77.5th, 85th, 92.5th, and 100th percentiles of the validation set's entropy distribution to define exclusion thresholds for the top 30%, 22.5%, 15%, 7.5%, and 0% most uncertain predictions, respectively. These entropy thresholds were then directly applied to the test set to exclude high-uncertainty samples. Performance metrics were calculated on the remaining, more confident test samples and aggregated across folds.

#### 2.4.2 Conformal prediction

To quantify the reliability of model predictions, we employed inductive conformal prediction, a model-agnostic framework that produces prediction sets with guaranteed error bounds under minimal assumptions. In our study, we targeted a 95% confidence level by setting the significance level to α = 0.05. The conformal prediction process was carried out within each cross-validation fold.

The procedure involved three main steps. First, we designated the validation set within each fold as a calibration set and applied the trained model to obtain predicted probabilities for the true class labels. From these, we computed nonconformity scores to quantify how well each calibration sample conformed to the model's predictions—lower scores indicating higher model confidence.

Second, we used the distribution of nonconformity scores from the calibration set to determine a threshold value corresponding to the desired confidence level. This threshold served as a cutoff for defining conformity during inference.

Finally, for each test sample, we computed class-conditional nonconformity scores (Papadopoulos et al., 2008) and formed a prediction set containing all classes that satisfied the conformity threshold. A prediction was labeled as certain if the prediction set contained exactly one class label, and uncertain if it contained either none or both labels.

The full mathematical formulation is provided in the Supplementary material.

## 3 Results

### 3.1 Results from machine learning models

**Figure 2:**
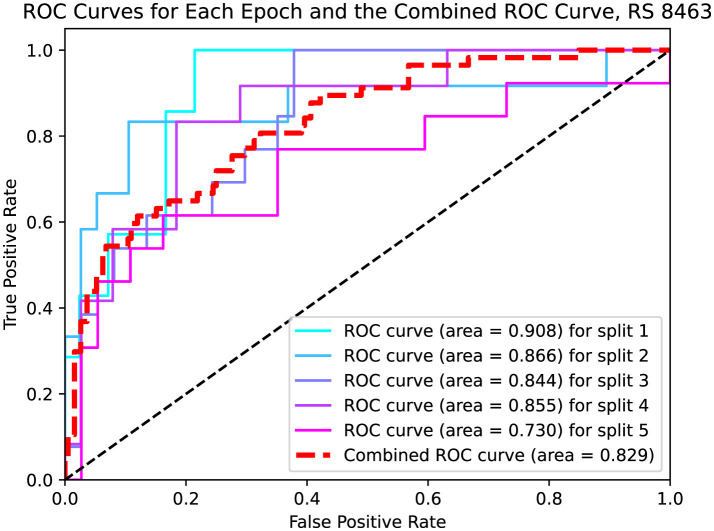
ROC curves for each epoch and the combined curve for RS 8463. Five individual curves are shown with different colors, each representing a data split with specific AUC values ranging from 0.730 to 0.908. The combined ROC curve is depicted in red with an AUC of 0.829. The graph plots true positive rate against false positive rate.

The ROC curves and AUC scores for each fold, along with the aggregated results across all folds using both pre- and post-treatment CT and PET images, are presented in Figure 2. In this case, the overall sensitivity is 0.614, while the overall specificity is 0.838. The combined AUC is 0.829 and the AUC for each fold ranges from 0.730 to 0.909. Out of the total test samples, 161 were true negatives, 22 were false positives, 31 were false negatives, and 35 were true positives.

Table 1 shows the results from model selection for a specific fold. Based on the selection criteria, the XGBoost model was chosen as the optimal model.

**Table 1:** Performance metrics of models based on various feature selection and cross-validation methods for a single fold are presented.

**FS method**	**Model**	**Accuracy**	**PRAUC**	**AUC**	**Sensitivity**	**Specificity**	**NPV**	**PPV**
LASSO	LR	0.8	0.493	0.717	0.538	0.892	0.846	0.636
LASSO	SVC	0.72	0.407	0.449	0.230	0.892	0.767	0.429
LASSO	SVCP	0.72	0.576	0.576	0.385	0.838	0.795	0.455
LASSO	EBC	0.82	0.600	0.790	0.462	0.946	0.833	0.75
LASSO	XGB	0.84	0.596	0.800	0.769	0.865	0.914	0.667

In this case, XGBoost achieved the highest Youden Index (1.634), making it the optimal model for this fold.

FS, feature selection; PRAUC, precision-recall area under the curve; AUC, area under the curve; NPV, negative predictive value; PPV, positive predictive value; LR, logistic regression; SVC, support vector classifier; SVC, support vector classifier with polynomial kernel; EBC, explainable boosting classifier; XGB, XGBoost.

We collected the test metrics from running the model with different random states, and the results are summarized in Table 2. The first five rows indicate the optimal model selected for each fold, with LR and XGBoost being the most frequently chosen. The last four rows present the key overall performance metrics, along with their mean and standard deviation on the right. Across five repeated runs, the model achieved a mean accuracy of 0.797 (95% CI: 0.780–0.813), AUC of 0.820 (95% CI: 0.800–0.840), sensitivity of 0.608 (95% CI: 0.586–0.631), and specificity of 0.852 (95% CI: 0.826–0.878).

**Table 2:** Models and decision thresholds selected as the best-performing for each fold across different random states (top five rows), alongside performance metrics: accuracy, AUC, sensitivity, and specificity (middle four rows).

**Metric / Fold**	**Random state 8,463**	**Random state 7,053**	**Random state 2,194**	**Random state 4,727**	**Random state 3,929**	**Mean ±SD**
Fold 1 best model	XGB, 0.31	LR, 0.23	XGB, 0.31	XGB, 0.24	LR, 0.27	–
Fold 2 best model	LR 0.2	LR 0.2	XGB, 0.2	LR, 0.21	LR, 0.22	–
Fold 3 best model	LR, 0.25	LR, 0.35	XGB, 0.5	XGB, 0.24	XGB, 0.24	–
Fold 4 best model	XGB, 0.2	XGB, 0.2	XGB, 0.25	LR, 0.3	XGB, 0.2	–
Fold 5 best model	LR, 0.38	LR, 0.21	XGB, 0.2	LR, 0.43	LR, 0.47	–
Accuracy	0.787	0.787	0.819	0.791	0.799	0.797 ± 0.012
AUC	0.829	0.797	0.838	0.823	0.813	0.820 ± 0.015
Sensitivity	0.614	0.632	0.586	0.614	0.596	0.608 ± 0.017
Specificity	0.839	0.833	0.885	0.843	0.859	0.852 ± 0.019
Importance clinical vs. radiomic	64.8%: 35.2%	77.0%: 23.0%	43.2%: 56.0%	62.2%: 37.8%	61.5%: 38.5%	-

The final row summarizes the average proportion of feature importance attributed to clinical and radiomic variables across folds. The last column summarizes the mean and standard deviation of the performance metrics.

### 3.2 Results from SAM-Med3D

The SAM-Med3D image encoder was evaluated using its pretrained weights and the checkpoints corresponding to the highest validation AUC achieved on the validation sets. The resulting performance metrics were as follows: Accuracy of 0.722, AUC of 0.658, Sensitivity of 0.525, and Specificity of 0.781. The test AUC values obtained from five-fold cross-validation were 0.642, 0.699, 0.684, 0.609, and 0.877, respectively.

### 3.3 Uncertainty analysis

**Figure 3:**
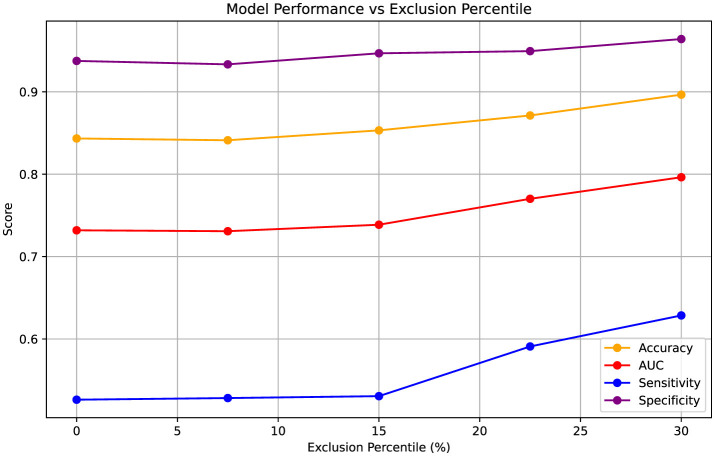
Variation in performance metrics (Accuracy, AUC, Sensitivity, Specificity) as increasingly uncertain test samples are excluded based on entropy percentiles. Entropy thresholds were computed from the validation sets and applied to the test sets at five levels of uncertainty exclusion (approximately 0%, 7.5%, 15%, 22.5%, and 30%). The results are aggregated over five-fold cross-validation, with the 0% exclusion level representing performance across all 249 test samples. Metrics are presented as point estimates.

After excluding test samples based on entropy values derived from predicted probabilities, we obtained five sets of performance metrics corresponding to exclusion thresholds at ~0%, 7.5%, 15%, 22.5%, and 30% of the most uncertain samples. These thresholds were determined from the validation sets and then directly applied to the test sets. The results, aggregated over five-fold cross-validation, are summarized in Figure 3. At the 0% exclusion level, all 249 samples served as test data across the folds. As increasingly uncertain samples were excluded, overall test performance improved consistently. Notably, sensitivity exhibited a marked increase-from 0.526 to 0.629-indicating better detection of true positive cases among the retained, more certain predictions.

The model performance on the certain and uncertain cohorts are shown in Table 3. Notably, the uncertain cohort includes a higher proportion of patients with failure. This suggests that these cases may inherently involve greater clinical complexity. Despite this, the prediction AUC for the uncertain cohort remains relatively high at 0.787.

**Table 3:** Performance and distribution metrics for certain and uncertain cohorts are presented, with cohort classification determined based on a significance level of α = 0.05.

**Metric**	**Certain cohort**	**Uncertain cohort**
Total patients	112	137
Successes (0)	95 (84.8%)	97 (70.8%)
Failures (1)	17 (15.2%)	40 (29.2%)
Accuracy	0.911	0.781
AUC	0.899	0.787
Sensitivity	0.529	0.525
Specificity	0.979	0.887

The samples with *p*-values below α were classified as uncertain, and those above were considered certain. Categorical outcomes are expressed as counts and percentages (*N*, %), and performance metrics (Accuracy, AUC, Sensitivity, Specificity) are represented as point estimates. A *p*-value < 0.05 was considered statistically significant. The certain cohort exhibits higher performance metrics, while the uncertain cohort also demonstrates relatively strong performance. Additionally, the uncertain cohort contains a higher proportion of cases with local failures (label 1).

## 4 Discussion

In this study, we proposed a predictive modeling framework for assessing local recurrence risk in HNSCC using paired pre- and post-treatment PET/CT imaging. Its strength lies in a structured model ensemble strategy: we systematically constructed a diverse set of classical machine learning models—varying by feature selection and algorithm type—and selected the best-performing model in each fold using a data-driven criterion. This multi-model approach was designed to mitigate the risk of overfitting or model instability, ensuring that if one model underperforms on a given split, others can compensate. We further validated this robustness by repeating the pipeline across multiple random data splits, consistently achieving high performance (mean AUC = 0.820, standard deviation = 0.015). A Vision Transformer-based model (SAM-Med3D) was also incorporated as an independent modeling path to diversify the overall predictive structure and evaluate deep learning approaches under the same task.

Integrating clinical data with radiomic features has been shown to enhance predictive performance in head and neck cancer recurrence studies. For instance, a study by Gangil et al. (2022) demonstrated that combining clinical and radiomic data using a Kernel Support Vector Machine (KSVM) significantly improved the prediction of locoregional recurrences in HNSCC patients. Similarly, Furukawa et al. developed Cox proportional hazard models that incorporated both clinical variables and multimodal radiomics features extracted from tumor regions in CT and PET images, achieving a concordance index of 0.74—outperforming the model that relied solely on clinical data (C-index of 0.67). Wang K. et al. (2023) further reinforced this finding, showing that integrating clinical and radiomic features improved predictive accuracy while leveraging a combination of SVM, Discriminant Analysis (DA), and Logistic Regression (LR). Motivated by these findings, we incorporated clinical data into our radiomics-based models, which resulted in strong predictive performance. In a complementary line of research, Eralp and Sefer proposed a reference-free transcriptomic analysis method in single-cell cancer data, emphasizing the potential of data-driven approaches in cancer characterization (Eralp and Sefer, 2024).

The most important clinical features selected by our models included Clinical T stage, smoking status, total fractions received, p16 status, and prescribed total radiation therapy (RT) dose. These variables are clinically relevant for predicting HNSCC recurrence, as they reflect both tumor burden and treatment intensity. Clinical T stage provides a measure of primary tumor extent, which strongly influences recurrence risk. Smoking status is a well-established prognostic factor associated with poorer outcomes and higher recurrence rates. Total fractions received and prescribed RT dose indicate the intended and delivered treatment intensity; deviations from the prescribed regimen could reflect treatment interruptions or modifications, potentially compromising effectiveness. Lastly, p16 status serves as a surrogate marker for HPV association, with p16-positive tumors typically showing better response to therapy and improved prognosis. Collectively, these features capture key clinical dimensions that influence recurrence risk and reinforce the interpretability of our model outputs.

The prescribed radiation doses and received radiation doses are similarly important, as discrepancies in these doses may suggest under-treatment or failure to complete treatment, potentially leading to tumor regrowth. Age is another key factor, as it is a well-known clinical prognostic factor for cancer outcomes, especially given that older adults often have greater comorbidities (Cadoni et al., 2017). Interestingly, Raab et al. (2024) found that older adults with head and neck cancer had higher rates of receiving inadequate radiation doses and are more likely to prematurely terminate treatment, which could lead to higher recurrence rates.

Most studies applying machine learning to radiomics data develop multiple types of models. For example, Fatima et al. (2021) employed quantitative ultrasound delta-radiomics with SVM and k-Nearest Neighbors (KNN), while Kim et al. (2022) utilized a LASSO and Logistic Regression (LR) model based on apparent diffusion coefficient maps from MRI. Wang et al. (2020) constructed SVM, Discriminant Analysis (DA), and LR models, combining their outputs through a weighted sum to generate the final prediction. Each of these approaches yielded promising results, with AUCs ranging from 0.75 to 0.8 on their respective datasets. In our study, we leveraged the advantages of EBC and XGBoost. EBC's iterative boosting process adaptively refines weak learners while maintaining interpretability, whereas XGBoost effectively captures complex nonlinear relationships through its regularized, scalable tree-boosting framework. Among five candidate models (LR, SVC, polynomial SVC, EBC, and XGBoost), the optimal model for each fold was selected based on performance. The impact of introducing EBC and XGBoost was evident: they were chosen as the best-performing model in 12 out of 25 cases. A study by Azeroual et al. (2024) also demonstrated that XGBoost performed well in predicting breast cancer recurrence, achieving good precision, recall, and F1 scores. Importantly, we employed cross-validation to ensure a comprehensive performance evaluation and conducted multiple runs with different random states to assess robustness. This approach ensures that model performance is not overly dependent on a specific data split or initialization, making the results more reliable.

The predictive models developed in this study have the potential to be highly valuable in the clinical setting by providing personalized risk evaluation for patients with HNSCC. These models could assist clinicians in identifying patients at higher risk for recurrence, allowing for more individualized treatment strategies. Our best-performing model achieved an AUC of 0.820, with a specificity of 0.852 and a sensitivity of 0.608—results that are strong by machine learning standards. However, we acknowledge that these metrics remain inferior to human performance using PET/CT surveillance, which has demonstrated sensitivities of 95%–96% and negative predictive values as high as 96% (Kim et al., 2013; Mehanna et al., 2016; Sheikhbahaei et al., 2015). While our model is not yet ready for clinical implementation, it represents a scalable and promising foundation for recurrence risk stratification, particularly in settings where imaging or specialist access may be limited.

We observed that the classical machine learning models outperformed the Vision Transformer-based model, despite the latter's more complex architecture and direct application to image data. This outcome may be attributed to several factors. Firstly, transformer models typically require large-scale datasets to effectively learn and generalize. Ma et al. (2022) explored the application of transformer models in medical imaging and highlighted that their performance is often limited by the relatively small size of medical datasets. Given that our dataset is relatively small, this limitation likely contributed to the suboptimal performance of the transformer model. Additionally, as Heidari et al. (2023) pointed out in their study, where they introduced a hybrid model combining convolutional neural networks (CNNs) and transformers, transformers excel at capturing long-range dependencies in medical images but often struggle to extract fine-grained, localized features. Because tumors are often small in size, this limitation may have reduced the Vision Transformer's ability to effectively distinguish their characteristics. Moreover, our approach used bounding boxes around the tumors to reduce input size and concentrate on the most relevant regions for analysis. While effective for focusing on the tumor itself, this strategy may limit the model's access to contextual information from the surrounding anatomy.

While transformer-based models offer architectural flexibility and the ability to model complex dependencies, their performance in this study was limited—likely due to factors such as small dataset size, restricted contextual input, and the localized nature of the target structures. Future improvements may be achieved through strategies such as hybrid CNN-transformer architectures to better capture local features (Lan et al., 2025), the use of larger input fields to preserve anatomical context, and the incorporation of pretraining or regularization techniques to reduce overfitting. Nonetheless, our primary objective was not to benchmark architectural complexity but to identify models that demonstrated strong and reliable performance in cross-validation. In this setting, classical models that combined handcrafted radiomic features with static clinical variables consistently outperformed the Vision Transformer and were selected for downstream analysis. These results align with previous findings Dai et al. (2021), which highlight the challenges of applying transformer-based models in data-constrained medical imaging scenarios.

Accurate uncertainty quantification is essential when deploying predictive models in high-stakes domains like healthcare. To this end, we used Test-Time Augmentation (TTA) to assess prediction uncertainty by introducing input perturbations and measuring entropy. As shown in Figure 3, excluding high-entropy samples led to consistent performance gains, particularly in AUC and sensitivity. We also employed conformal prediction to stratify model outputs into "certain" and "uncertain" cohorts, revealing that patients in the uncertain group exhibited a higher incidence of recurrence. This suggests that predictive uncertainty may correspond to more complex or atypical clinical presentations and could provide actionable insights for clinical practice. For instance, identifying patients with high uncertainty could prompt more intensive follow-up, potentially including additional imaging (e.g., MRI) or earlier follow-up assessments. Moreover, such stratification could inform multidisciplinary discussions, helping clinicians tailor follow-up strategies beyond standard protocols. It is important to emphasize that the model is intended as an assistive tool, not a standalone diagnostic system; ultimately, the decision-making authority rests with the treating physician, who will incorporate these outputs alongside other clinical information. Our findings align with prior studies, including the review by Vazquez and Facelli (2022), which highlights the utility of conformal prediction in providing valid, individualized confidence measures in clinical settings. Integrating uncertainty quantification into predictive models may thus enhance their clinical trustworthiness and facilitate more proactive decision-making.

Despite the promising findings, this study has several limitations. First, the dataset size remains limited. A larger dataset would allow for more comprehensive validation and improved model performance. While the current models have demonstrated strong predictive capabilities, their external applicability remains untested. We acknowledge that external validation on independent datasets is essential before clinical deployment, as it is necessary to ensure generalizability and robustness across different patient populations and imaging protocols. Future work should evaluate these models on independent external datasets. Second, a substantial proportion of patients had unknown HPV and p16 status. This missing information may have limited the model's ability to fully capture relevant recurrence patterns. Lastly, the Vision Transformer-based model may benefit from further hyperparameter tuning, alternative architectural configurations to improve predictive performance, or the integration of clinical features. Additional experiments optimizing its structure and training strategies could lead to improved results. Nonetheless, its inclusion in this study was purposeful: our overarching goal was to evaluate a diverse set of models and select the most effective one based on empirical validation performance. While the transformer did not outperform simpler classical models, this outcome highlights the importance of data-driven model selection.

## 5 Conclusion

This study demonstrates the effectiveness of AI-driven models in predicting HNSCC local recurrence using radiomic and clinical features. Traditional machine learning models, particularly XGBoost and EBC, achieved strong predictive power. Selecting the optimal model from a diverse pool enhanced the overall model's robustness and stability. The integration of uncertainty quantification methods provided additional insights into model reliability and potential clinical applicability.

## Data Availability

The raw data supporting the conclusions of this article will be made available by the authors, without undue reservation.
