# Supplementary material for: Enhancing prediction of primary site recurrence in head and neck cancer using radiomics and uncertainty estimation
Source: Front Artif Intell. 2025 Sep 8;8:1623393. doi: 10.3389/frai.2025.1623393 (PMC12450971; doi:10.3389/frai.2025.1623393)
Supplement: Supplementary file 1 [file Presentation_1.pdf]

Supplementary Material

1 EVALUATION METRICS USED IN THE ANALYSIS

We evaluated model performance using several standard metrics, each defined mathematically as follows. Accuracy is the proportion of correctly classified samples and is computed as

$$\text{Accuracy} = \frac{TP + TN}{TP + TN + FP + FN}$$

where TP, TN, FP, and FNdenote true positives, true negatives, false positives, and false negatives, respectively. Sensitivity (also called recall) measures the proportion of actual positive cases correctly identified and is calculated as

$$\text{Sensitivity} = \frac{TP}{TP + FN}.$$

Specificity measures the proportion of actual negative cases correctly identified and is given by

$$\text{Specificity} = \frac{TN}{TN + FP}.$$

The positive predictive value (PPV), or precision, is defined as

$$\text{PPV} = \frac{TP}{TP + FP},$$

while the negative predictive value (NPV) is

$$\text{NPV} = \frac{TN}{TN + FN}.$$

To identify the optimal model in each fold, we also computed the Youden Index, defined as

$$\text{Youden Index} = \text{Sensitivity} + \text{Specificity} - 1.$$

We further report the area under the receiver operating characteristic curve (AUC), which summarizes the tradeoff between sensitivity and specificity across various classification thresholds. Additionally, we report the area under the precision-recall curve (PRAUC), which is particularly informative in imbalanced datasets, as it focuses on the model's performance in predicting the minority class.

2 MATHEMATICAL FORMULATIONS FOR UNCERTAINTY QUANTIFICATION

2.1 Off-Centered Entropy for Predictive Uncertainty Quantification

To quantify predictive uncertainty in a way that reflects the actual decision threshold used by the classifier, we adopted an off-centered entropy formulation. Traditional entropy-based uncertainty measures assume a symmetric threshold at $p = 0.5$, which may not be appropriate in settings involving class imbalance

or threshold optimization. Our approach adjusts for this by centering the entropy peak at the operating threshold τ, which is optimized on the validation set in each fold.

Let $p \in [0, 1]$ denote the predicted probability of the positive class, and let $\tau \in (0, 1)$ be the optimal decision threshold. We define a transformed probability π, which rescales the range $[0, 1]$ such that the value $\pi = 0.5$ aligns with $p = \tau$, as follows:

$$\pi(p) = \begin{cases} \dfrac{p}{2\tau}, & \text{if } 0 \leq p \leq \tau \\ \dfrac{p + 1 - 2\tau}{2(1 - \tau)}, & \text{if } \tau < p \leq 1 \end{cases}$$

Using this transformed value π, the off-centered entropy $\mathcal{H}(p)$ is calculated as:

$$\mathcal{H}(p) = -[\pi \log \pi + (1 - \pi) \log(1 - \pi)].$$

This formulation shifts the maximum entropy from the default threshold of 0.5 to the fold-specific decision boundary τ, resulting in a more accurate reflection of model uncertainty in the context of calibrated or imbalanced predictions. This method builds upon asymmetric entropy formulations and was used throughout our analysis to identify low-confidence predictions and assess the relationship between uncertainty and outcome.

2.2 Conformal Prediction: Mathematical Formulation

Conformal prediction is a framework for quantifying the reliability of model predictions by producing prediction sets that satisfy a user-defined confidence level under minimal distributional assumptions. In this study, we implemented inductive conformal prediction with a significance level of $\alpha = 0.05$, targeting a 95% confidence level. For each fold in cross-validation, the validation set was used as the calibration set.

The procedure begins by applying the trained model to the calibration set and recording the predicted probabilities for the true class labels. For each calibration sample i, we compute the nonconformity score:

$$s_i = 1 - \hat{p}_{y_i},$$

where $\hat{p}_{y_i}$ is the predicted probability assigned to the true label $y_i \in \{0, 1\}$ for sample i **?**. This score reflects the model's confidence in the correct class; higher values indicate lower conformity.

We then compute the $(1 - \alpha)$-quantile of the calibration-set nonconformity scores and denote it by κ. For each test sample, we evaluate its class-conditional conformity scores s_c, adjusted for the optimal decision threshold τ **?**. To do so, we define the normalized distance from τ as:

$$d(x; \tau) \; = \; \begin{cases} \dfrac{\tau - x}{\tau}, & x \leq \tau, \\ \dfrac{x - \tau}{1 - \tau}, & x > \tau, \end{cases}$$

Then, the class-conditional nonconformity scores are given by:

$$s_1 = 1 - d\left(\hat{p}_{y_1};\tau\right), \qquad s_0 = 1 - d\left(1 - \hat{p}_{y_1};\tau\right).$$

The prediction set for a sample consists of every class $c \in \{0, 1\}$ for which $s_c \leq \kappa$. A prediction is considered "certain" if the prediction set contains exactly one class label, and "uncertain" if the set contains zero or both labels. This approach provides a calibrated confidence measure that reflects not only the predicted probabilities but also their relation to the calibrated decision boundary.

3 SUPPLEMENTARY TABLES AND FIGURES

Variables	Count	Percentage
**Ethnicity**		
Caucasian	179	71.9%
African American	31	12.4%
Hispanic	25	10.0%
Asian	11	4.4%
Other	2	0.8%
Unknown	1	0.4%
**Gender**		
Male	198	79.5%
Female	51	20.5%
**Cancer Histology**		
Squamous cell carcinoma	238	95.6%
Adenoid cystic carcinoma	3	1.2%
Large Cell Carcinoma	1	0.4%
Other	7	2.8%
**Disease Site**		
Oropharynx	170	68.3%
Nasopharynx	30	12.0%
Oral cavity	25	10.0%
Larynx	11	4.4%
Hypopharynx	2	0.8%
Unknown primary	1	0.4%
**Clinical T Stage**		
TX	3	1.2%
T0	1	0.4%
T1	45	18.1%
T2	91	36.5%
T3	59	23.7%
T4	50	20.1%
**Clinical N Stage**		
N0	37	14.9%
N1	42	16.9%
N2	162	65.1%
N3	8	3.2%
**HPV Status**		
HPV positive	61	24.5%
HPV negative	53	21.3%
Unknown	135	54.2%
**p16 Status**		
p16 positive	101	40.6%
p16 negative	37	14.9%
Unknown	111	44.6%

Table S1. Demographic Characteristics of the Study Population

Variables	Count	Percentage
**Smoking Status**		
Never smoker	81	32.5%
Ever smoker	124	49.8%
Current smoker	44	17.7%
**Concurrent Chemo**		
Yes	215	86.3%
No	34	13.7%
**Prescribed Total RT Dose (cGy)**		
25th Percentile (Q1)	6600	—
Median	6800	—
75th Percentile (Q3)	7000	—
Standard Deviation	343.59	—
**Original Fractions**		
25th Percentile (Q1)	32	—
Median	33	—
75th Percentile (Q3)	35	—
Standard Deviation	2.23	—

Table S2. Continuation of demographic characteristics of the study population.

The following table lists the clinical features used in this study, along with a column for additional notes or descriptions.

Clinical Feature Name	Description
Cancer Histology	
Grade	Low vs high grade
Disease Site	
Clinical T Stage	Clinical radiation oncology T Staging
Clinical N Stage	Clinical radiation oncology N Staging
HPV Status	HPV status in patients with oropharyngeal cancer
p16 Status	p16 status in patients with oropharyngeal cancer
Smoking Status	
Treatment Paradigm	
Received Total RT Dose	
Completed Treatment	If patient completed treatment and received prescribed dose
Total Fractions Received	
Induction Chemo	
Concurrent Chemo	
Age RT Started	RT Start Date - DOB, an approximation of age at RT

Table S3. List of clinical features and their corresponding notes.
